# *Fusobacterium* *nucleatum*-Stimulated OSCC Cell-Derived Exosomes Induce a Pro-Adhesive Phenotype in Lymphatic Endothelial Cells *via* the ROS/NF-κB/ICAM1 Axis

**DOI:** 10.3390/microorganisms14091965

**Published:** 2026-09-05

**Authors:** Li Wei, Qi He, Haiting Gao, Wanheng Li, Tianyong Sun, Qiang Feng

**Affiliations:** 1 Department of Human Microbiome, School and Hospital of Stomatology, Cheeloo College of Medicine, Shandong University & Shandong Key Laboratory of Oral Diseases & Shandong Engineering Research Center of Dental Materials and Oral Tissue Regeneration & Shandong Provincial Clinical Research Center for Oral Diseases, Jinan 250012, China; 202221021@mail.sdu.edu.cn (L.W.);; 2 School of Stomatology, College of Life Sciences and Medicine, Northwest University, Xi’an 710069, China; 3 State Key Laboratory of Microbial Technology, Shandong University, Qingdao 266237, China; 4 Shandong University-BOP Joint Oral Microbiome Laboratory, Jinan 250012, China

**Keywords:** *Fusobacterium nucleatum*, oral squamous cell carcinoma, exosome, lymphatic endothelial cells, ICAM1

## Abstract

The role of *Fusobacterium nucleatum* (*F. nucleatum*) in regulating exosome-mediated communication between oral squamous cell carcinoma (OSCC) cells and lymphatic endothelial cells (LECs) remains poorly understood. Here, we confirmed the presence of *F. nucleatum* in OSCC tissues and showed that it induced inflammation- and vesicle-associated transcriptional changes in OSCC cells. Exosomes were isolated by differential ultracentrifugation, and quantitative analyses demonstrated that *F. nucleatum* stimulation increased exosome yield from OSCC cells. In vitro, pretreatment of LECs with exosomes derived from *F. nucleatum*-stimulated OSCC cells (Fn-Exo) enhanced OSCC cell adhesion to LEC monolayers. Fn-Exo upregulated intercellular adhesion molecule 1 (ICAM1) expression in LECs, and ICAM1 blockade partially reduced Fn-Exo-induced OSCC cell adhesion to LECs. Fn-Exo increased intracellular reactive oxygen species (ROS) accumulation and promoted NF-κB p65 phosphorylation and nuclear translocation in LECs. N-acetyl-L-cysteine (NAC) attenuated Fn-Exo-induced ROS accumulation and NF-κB activation, and both NAC and the NF-κB inhibitor BAY 11-7082 reduced ICAM1 upregulation and partially attenuated the enhanced adhesion of OSCC cells to Fn-Exo-treated LECs. These pharmacological inhibition experiments support the involvement of ROS/NF-κB/ICAM1 signaling in the Fn-Exo-induced pro-adhesive phenotype. Collectively, these findings indicate that *F. nucleatum* is associated with increased exosome yield from OSCC cells and that Fn-Exo enhances the adhesive interaction between OSCC cells and LECs.

## 1. Introduction

Oral squamous cell carcinoma (OSCC) is one of the most common malignancies of the head and neck region and shows a high propensity for cervical lymph node metastasis (LNM) [1,2], which is a major determinant of prognosis in patients with OSCC [3]. Accumulating evidence has highlighted the involvement of the oral microbiota in OSCC progression, among which *Fusobacterium nucleatum* (*F. nucleatum*) has emerged as a tumor-associated bacterium frequently linked to oral cancer development [4,5]. Specifically, *F. nucleatum* can modulate tumor cell-intrinsic phenotypes, including proliferation, migration, invasion, and immune-related responses [6,7,8]. *F. nucleatum* has been reported to accumulate at the invasive margins of OSCC tissues and to be more abundant in tumors with cervical LNM [9]. However, whether *F. nucleatum* remodels the lymphatic metastatic microenvironment through tumor–lymphatic endothelial interactions remains unclear.

Exosomes are key mediators of intercellular communication that transport proteins, lipids, nucleic acids, and other bioactive molecules to recipient cells [10,11]. Tumor-derived exosomes have increasingly been recognized as important regulators of tumor microenvironment remodeling, organotropic metastasis, and pre-metastatic niche formation [12,13]. In addition, *F. nucleatum* infection has been reported to alter the molecular cargo and increase the secretion of tumor cell-derived exosomes, thereby modulating the biological functions of recipient cells [14]. In OSCC, tumor cell-derived exosomes can be internalized by human lymphatic endothelial cells (LECs) and regulate lymphangiogenesis-related functions, including proliferation, migration, and tube formation [15,16]. OSCC-derived exosomes have also been reported to modulate endothelial cell functions under hypoxic conditions [17]. These findings raise the possibility that microbial cues in the OSCC microenvironment may reshape tumor-derived exosomes and thereby influence lymphatic endothelial behavior.

During LNM, lymphatic vessels and LECs are not merely passive conduits, but constitute an active cellular interface through which tumor cells interact with the lymphatic system and the metastatic microenvironment [18,19,20]. Tumor–LEC adhesion directly reflects the interaction between tumor cells and the lymphatic endothelium and represents an important aspect of lymphatic endothelial remodeling associated with metastatic progression. Tumor-derived exosomes have been shown to disseminate through the lymphatic system to sentinel or draining lymph nodes and be taken up by LECs [21]. Tumor-derived extracellular vesicles and soluble factors have been shown to promote a pro-adhesive endothelial phenotype, characterized by increased adhesion molecule expression and enhanced tumor cell adhesion [22,23]. ICAM1 is one of the major adhesion molecules involved in endothelial inflammatory responses and cell–cell interactions, and ICAM1-mediated interactions between cancer cells and LECs have been implicated in sentinel lymph node metastasis [24]. Together, these findings highlight the biological relevance of tumor–LEC adhesion in lymphatic metastatic progression.

In this study, we investigated whether exosomes derived from *F. nucleatum*-stimulated OSCC cells remodel the pro-adhesive state of LECs. After confirming the presence of *F. nucleatum* in OSCC tissues and defining transcriptional responses related to inflammation and vesicle trafficking in OSCC cells, we isolated and characterized exosomes from *F. nucleatum*-stimulated OSCC cells. We further assessed their effects on LEC adhesion-related phenotypes and explored the underlying molecular mechanism. Our study provides a cellular mechanism by which *F. nucleatum*-stimulated OSCC cells may remodel the adhesive state of LECs through exosome-mediated cell–cell communication.

## 2. Materials and Methods

### 2.1. Bacterial Annotation in OSCC

Raw RNA-sequencing data from OSCC samples were downloaded from the GEO database under the accession numbers GSE184616 (15 samples) and GSE275870 (108 samples). SRA files were converted into paired-end FASTQ files using SRA Toolkit (version 3.4.1), followed by quality control and adapter trimming using fastp (version 1.0.1). High-quality reads were processed using the GATK PathSeq pipeline (version 4.6.2.0). After removal of host-derived reads, the remaining reads were aligned against the Human Reference Oral Microbiome (HROM) database for taxonomic classification and microbial abundance estimation.

### 2.2. Ethics, Tissue Microarray, and Fluorescence In Situ Hybridization (FISH)

A human OSCC tissue microarray was purchased from Zhongke Guanghua Intelligent Biotechnology Co., Ltd. (Xi’an, China, https://www.bioaitech.com/). Use of clinical samples was approved by the Ethics Committee and Institutional Review Board of the School and Hospital of Stomatology, Cheeloo College of Medicine, Shandong University in compliance with ethical standards and patient confidentiality (approval no. 20240923; approval date: 26 September 2024). Informed consent was obtained from all patients regarding the use of tissue samples.

A FITC-conjugated *F. nucleatum* probe with the sequence 5′-CGCAATACAGAGTTGAGCCCTGC-3′ was used to detect the colonization and distribution of *F. nucleatum* in OSCC tissue microarrays. Following rehydration and deparaffinization, all specimens were treated with proteinase K and 0.2 N HCl for 10 min to enhance probe penetration. After incubation in a blocking buffer for 2 h at 55 °C, the probe (diluted 1:50 in 35% preheated hybridization buffer for 3 min at 88 °C) was added and hybridized overnight at 42 °C in a humid, dark chamber. The specimens were then washed in Tris-HCl buffer (20 mM, pH 7.2) containing 40 mM NaCl to remove unbound probes and mounted using a DAPI–antifade solution. Fluorescent images were captured using LSM980 confocal laser scanning microscope (CLSM) (Zeiss, Jena, Germany).

### 2.3. Culture of Bacterial Strains

*F. nucleatum* ATCC 25586 was cultured in a modified brain heart infusion broth medium (BHI; BD Biosciences, Sparks, MD, USA) supplemented with 5 μg/mL hemin and 1 μg/mL vitamin K1 under anaerobic conditions. The bacterial concentration was standardized to an optical density (OD) of 1.0 at 600 nm using a spectrophotometer, corresponding to 2 × 10^8^ bacteria/mL. *E. coli* strain DH5α (Tiangen, Beijing, China) was cultured in Luria–Bertani (LB) broth overnight at 37 °C with shaking. The concentration of *E. coli* DH5α was adjusted to an OD_600_ of 1.0, corresponding to 8 × 10^8^ bacterial cells/mL.

### 2.4. Cell Culture and Bacteria–Cell Coculture

The human OSCC cell lines CAL27 and SCC15, as well as human lymphatic endothelial cells (LECs), were purchased from American Type Culture Collection (ATCC). CAL27 cells were cultured in high-glucose DMEM (Gibco, Suzhou, China), SCC15 cells were cultured in DMEM/F12 (Gibco, China), and LECs were cultured in endothelial cell growth medium (Gibco, China). All media were supplemented with 10% FBS (Gibco, China) at 37 °C in a humidified incubator containing 5% CO_2_. For bacteria–cell coculture experiments, OSCC cells were seeded in culture plates and grown to approximately 60–70% confluence. Before bacterial stimulation, cells were washed with PBS and cultured in antibiotic-free medium. Logarithmic-phase *F. nucleatum* or *E. coli* was added to OSCC cells at the indicated multiplicity of infection (MOI). *E. coli* was included as a nonpathogenic bacterial comparator to control for nonspecific responses to bacterial exposure. For the untreated control groups, cells were treated with an equal volume of sterile medium.

### 2.5. Cell Apoptosis Assay

Cell apoptosis was analyzed using an Annexin V-FITC/propidium iodide (PI) apoptosis detection kit (Beyotime, Shanghai, China) according to the manufacturer’s instructions. Briefly, OSCC cells were treated with *F. nucleatum* (MOI of 10, 50, 100) or medium control. After 48 h, both floating and adherent cells were collected, washed twice with cold PBS, and resuspended in binding buffer. Cells were then stained with Annexin V-FITC and PI for 15 min at room temperature in the dark. After staining, samples were analyzed using BD Accuri C6 Plus flow cytometer (BD Biosciences, San Jose, CA, USA). Data were analyzed using FlowJo software v10. An MOI of 50 was selected for subsequent experiments based on the apoptosis assessment at different MOIs.

### 2.6. RNA-Sequencing

CAL27 cells were seeded in tissue culture plates at a density of 5 × 10^5^ cells per well and stimulated with *F. nucleatum* at a MOI of 50 for 48 h. Total RNA was subsequently extracted using TRIzol reagent (AG, Beijing, China). The experiments were independently carried out three times for each group. RNA-sequencing was performed by Wuhan HuaDaGene Institute (Wuhan, China).

Differentially expressed genes (DEGs) were identified using the limma-trend method implemented in the edgeR (version 4.10.1) and limma (version 3.68.3) R packages. Raw *p* values were adjusted for multiple comparisons using the Benjamini–Hochberg method. Genes with an adjusted *p* value < 0.05 and a fold change > 1.5 were considered differentially expressed. The DEG results were visualized by generating a volcano plot using the R package ggplot2 (version 4.0.3) and a heatmap using the R package ComplexHeatmap (version 2.28.0). GO and KEGG enrichment analyses of the obtained DEGs were performed using the R package clusterProfiler (version 4.20.0). *p* < 0.01 and adjusted *p* < 0.05 were used to detect the statistically significant items. Selected enrichment results were visualized as dot plots using the R package ggplot2. GSEA analysis was performed using the R package clusterProfiler based on the results from the differential analysis. The ordered rank metric was used as the input, calculated using the function sign(logFC) * −log_10_(*p* value + 1 × 10^−10^). Hallmark gene sets used as the reference gene list (h.all.v2023.2.Hs.symbols.gmt) were downloaded from the Molecular Signatures Database (MSigDB, https://www.gsea-msigdb.org/gsea/msigdb, accessed on 20 June 2026). |NES| > 1, *p* < 0.05, and adjusted *p* < 0.25 were used to select the statistically significant GSEA results.

### 2.7. Exosome Extraction

OSCC cells were stimulated with *E. coli* or *F. nucleatum* for 48 h at an MOI of 50. Then, cells were washed with PBS, and the cell culture medium was replaced with the corresponding fresh culture medium containing 10% exosome-depleted FBS, followed by an additional 48 h of culture for exosome collection, as previously described [25,26]. After centrifugation at 2000 *g* for 15 min to remove cell debris, conditioned media were centrifuged at 10,000 *g* for 60 min at 4 °C. The supernatant was then filtered through a 0.45 μm filter and a 0.22 μm filter and ultracentrifuged at 120,000 *g* for 120 min at 4 °C. The exosome pellet was washed with sterile PBS and ultracentrifuged again at 120,000 *g* for 120 min. The final exosome pellet was resuspended in sterile PBS for subsequent experiments. Three independent exosome preparations were obtained for each experimental group.

### 2.8. Exosome Characterization

The morphology of isolated exosomes was examined by TEM. Briefly, 20 μL of exosome suspension was placed onto a copper grid and allowed to adsorb for approximately 10 min. Excess liquid was removed with filter paper, and the grid was negatively stained with 2% phosphotungstic acid solution for 3 min. After air drying, exosomes were observed and photographed using a JEM1400 transmission electron microscope (JEOL, Tokyo, Japan). Particle size distribution and concentration of isolated exosomes were measured using a ZetaView PMX120 nanoparticle tracking analyzer (Particle Metrix, Inning am Ammersee, Germany). Exosome samples were diluted in sterile ddH_2_O to achieve an appropriate particle concentration for measurement. Particle size distribution and particle concentration were analyzed using ZetaView software (version 8.05.12 SP2). The total protein content of samples was measured using a BCA Protein Assay Kit (Beyotime, China; P0009). The particle-to-protein ratio (particles/μg protein) was calculated as an additional purity-related parameter [27]. Western blotting was performed using 10 μg of exosomal protein to detect exosome-enriched proteins (HSP70, TSG101, and CD63) and the nuclear marker histone H3, which was used to assess potential nuclear contamination [28].

### 2.9. Exosome Uptake Assay

Purified exosomes were labeled with lipophilic fluorescent dye PKH26 (MedChemExpress, South Brunswick Township, NJ, USA) for visualization. Untreated LECs served as the control, and a dye-only negative control without exosomes was included to assess nonspecific PKH26 fluorescence. PKH26-labeled exosomes (10 μg) were co-cultured with 1 × 10^5^ LECs for 1, 3, 6, 12, and 24 h. After washing with PBS, the cells were collected for flow cytometry analysis (BD Biosciences, San Jose, CA, USA) or LSM980 CLSM observation (Zeiss, Jena, Germany) to determine the uptake of exosomes by the recipient cells.

### 2.10. Adhesion Assay

LECs were seeded in 96-well plates and grown overnight to reach full confluence. After pretreatment with exosomes (10 μg per 1 × 10^5^ LECs) for 12 h, 5 × 10^4^ PKH26-labeled CAL27/SCC15 cells were washed three times with PBS, resuspended in serum-free culture medium, and add to the monolayer of LECs, and the mixture was co-cultured at 37 °C for 15 min, as previously described [29]. After washing with PBS gently three times to remove the non-adherent cells, the adherent tumor cells in the monolayers were observed and imaged with a fluorescence microscope. Each experiment was analyzed in triplicate. For ICAM1 blockade experiments, LECs were preincubated with an anti-ICAM1 neutralizing antibody (R&D Systems, Minneapolis, MN, USA, AF720) at 12.5 μg/mL for 1 h before the addition of PKH26-labeled OSCC cells. An isotype-matched IgG antibody (R&D Systems, 5-001-A) was used as a negative control. To inhibit NF-κB signaling, LECs were pretreated with BAY 11-7082 (5 μM) for 1 h prior to Fn-Exo treatment. The number of adherent PKH26-positive tumor cells was quantified from three randomly selected fields using ImageJ software version 1.54.

### 2.11. Reverse Transcription-Quantitative Polymerase Chain Reaction (RT-qPCR)

RNA was extracted using AG RNAex Pro RNA Reagent (Accurate Biotechnology Co., Ltd., Changsha, China; AG21102) and converted to cDNA using an Evo M-MLV RT Mix Kit (Accurate Biotechnology Co., Ltd., China; AG11728). RT-qPCR was performed using a Pro Taq HS Premix Probe qPCR Kit (Accurate Biotechnology Co., Ltd., China; AG11730). For the two-step protocol, genomic DNA was first removed by incubating RNA samples with gDNA Clean Reagent at 42 °C for 2 min, followed by reverse transcription for cDNA synthesis. RT-qPCR was performed using 2 × Pro Taq HS Probe Premix under the following conditions: initial denaturation at 95 °C for 30 s, followed by 40 cycles of denaturation at 95 °C for 5 s and combined annealing/extension at 60 °C for 30 s. Three biological replicates were prepared from independent cell samples. Relative gene expression levels were calculated using the 2^−ΔΔCt^ method. All primer sequences used in this study are listed in Supplementary Table S1.

### 2.12. Western Blotting

Cells were lysed using RIPA buffer containing protease and phosphatase inhibitor cocktails (both at a 1:100 dilution) for 15 min on ice, then centrifuged for 15 min at 4 °C and 12,000 rpm. Equal amounts of protein for each sample (20–30 µg) were subjected to SDS-PAGE, then transferred to nitrocellulose membranes. The primary antibodies used include HSP70 (1:50,000, Proteintech, Rosemont, IL, USA, 66183-1-Ig), TSG101 (1:10,000, Proteintech, 28283-1-AP), CD63 (1:20,000, Proteintech, 25682-1-AP), Histone H3 (1:10,000, Proteintech, 17168-1-AP), ICAM1 (1:5000, Proteintech, 10831-1-AP), Phospho-NF-κB p65 (1:5000, Proteintech, 82335-1-RR), NF-κB p65 (1:5000, Proteintech, 80979-1-RR), and GAPDH (1:50,000, Proteintech, 60004-1-Ig). The horseradish peroxidase (HRP)-conjugated secondary antibodies used were from Proteintech (1:10,000, Rabbit SA00001-2 and Mouse SA00001-1), and the ECL substrate was detected using an Omni-ECL™ enhanced chemiluminescence detection kit (Epizyme, Shanghai, China; SQ201).

### 2.13. Reactive Oxygen Species (ROS) Staining

To determine whether NAC attenuates Fn-Exo-induced ROS production, LECs were incubated in the absence or presence of NAC (5 mM) for 1 h and then treated with Fn-Exo (10 μg per 1 × 10^5^ LECs) for 12 h before ROS detection. ROS levels in LECs were detected using the fluorescent probe DCFH-DA (Beyotime, China; S0033S) according to the manufacturer’s instructions. Briefly, cells were washed with PBS and incubated with 10 μM DCFH-DA for 30 min at 37 °C in the dark. After staining, cells were washed three times with serum-free medium or PBS to remove excess probe. Fluorescence signals were observed using a fluorescence microscope under identical exposure settings, and quantitative analysis was performed using ImageJ software version 1.54.

### 2.14. Immunofluorescence (IF)

LECs were seeded into confocal dishes and treated as indicated. Cells were washed with PBS and fixed with 4% paraformaldehyde for 10 min. After permeabilization with 0.1% Triton X-100 for 10 min, cells were blocked with 5% BSA or normal goat serum for 1 h at room temperature. Cells were incubated with primary antibodies against NF-κB p65 (1:250, Proteintech, 80979-1-RR) overnight at 4 °C. After washing, cells were incubated with fluorescence-conjugated secondary antibodies for 1 h at room temperature in the dark. Nuclei were counterstained with DAPI. The fluorescence intensity of p65 in the nuclear and cytoplasmic compartments was quantified using ImageJ software version 1.54.

### 2.15. Statistical Analysis

Statistical analyses were performed using GraphPad Prism 9.5. Data are presented as mean ± standard deviation (SD) from at least three independent biological replicates. Prior to parametric analysis, data were evaluated for normality using the Shapiro–Wilk test and for equality of variances using the Brown–Forsythe test. For datasets satisfying these assumptions, comparisons between two groups were performed using an unpaired Student’s *t*-test. For comparisons among multiple groups, one-way analysis of variance (ANOVA) was conducted, followed by Dunnett’s post hoc test for comparisons against a control group, Tukey’s post hoc test for pairwise comparisons, or Sidak’s post hoc test selected pairwise comparisons, as appropriate. Statistical significance was established at two-sided *p* < 0.05.

## 3. Results

### 3.1. F. nucleatum Induces an Inflammatory Transcriptional Program in OSCC Cells

**Figure 1:**
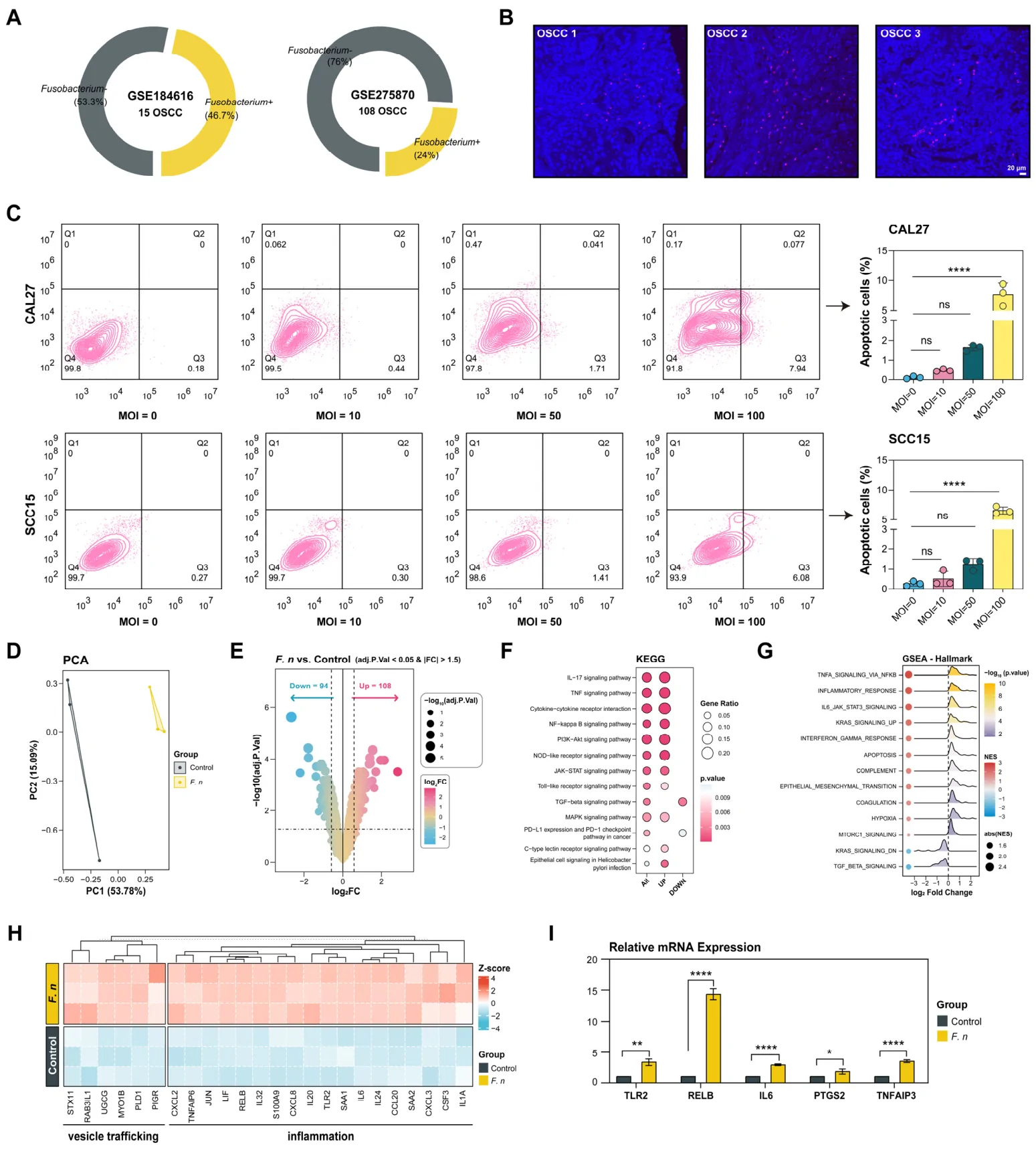
*F. nucleatum* induces an inflammatory transcriptional program in OSCC cells. (**A**) Distribution of OSCC samples with (yellow) and without (gray) detectable *Fusobacterium* reads in the GSE184616 (*n* = 15) and GSE275870 (*n* = 108) datasets. (**B**) Representative FISH images showing *F. nucleatum* in OSCC tissues. Scale bar, 20 μm. (**C**) OSCC cells were stimulated with *F. nucleatum* (at MOIs of 10, 50, and 100), and apoptosis was assessed by Annexin V/PI flow cytometry. Representative flow cytometry plots and quantitative analysis of apoptotic cells are shown (*n* = 3). (**D**) PCA of RNA-seq data from control and *F. nucleatum*-stimulated OSCC cells. (**E**) Volcano plot showing differentially expressed genes between control and *F. nucleatum*-stimulated OSCC cells. (**F**) KEGG pathway enrichment analysis of the DEGs. (**G**) GSEA of control and *F. nucleatum*-stimulated OSCC cells. (**H**) Heatmap showing the expression patterns of representative inflammation- and vesicle trafficking-related genes. (**I**) RT-qPCR validation of selected inflammation-related genes (*n* = 3). Data are presented as mean ± SD. Statistical significance was determined by one-way ANOVA followed by Dunnett’s multiple-comparisons test in (**C**) and two-tailed unpaired Student’s *t*-test in (**I**). ns, not significant; * *p* < 0.05, ** *p* < 0.01, **** *p* < 0.0001.

To assess the frequency of the *Fusobacterium* genus, we analyzed microbial reads from two independent OSCC RNA-seq datasets. *Fusobacterium* reads were detected in 46.7% (7/15) of samples from GSE184616 and 24.0% (26/108) of samples from GSE275870 (Figure 1A). Pairwise correlation analysis further revealed positive associations between *Fusobacterium* and several bacterial genera previously implicated in human cancers (Figure S1A). At the species level, FISH using an *F. nucleatum*-specific probe further confirmed the presence of *F. nucleatum* in OSCC tissues (Figure 1B). To determine an appropriate bacterial dose for subsequent experiments, cell apoptosis was assessed across different MOIs in vitro, and an MOI of 50 was selected because it did not significantly induce apoptosis (Figure 1C). Cell counting showed an increase in CAL27 and SCC15 cell numbers following *F. nucleatum* stimulation for over 48 h (Figure S3). To comprehensively characterize the transcriptional changes induced by *F. nucleatum* in OSCC cells, RNA-seq analysis was performed. Principal component analysis (PCA) showed clear separation between the control and *F. nucleatum* groups (Figure 1D). Volcano plot analysis identified 202 differentially expressed genes in *F. nucleatum*-stimulated OSCC cells, including 108 upregulated and 94 downregulated genes (adjusted *p* < 0.05 and |FC| > 1.5) (Figure 1E, Table S2). Kyoto Encyclopedia of Genes and Genomes (KEGG) pathway enrichment analysis showed that the DEGs were mainly enriched in the TNF, NF-κB, NOD-like receptor, and Toll-like receptor signaling pathways (Figure 1F). Consistently, gene set enrichment analysis (GSEA) revealed positive enrichment of the TNFA_SIGNALING_VIA_NFKB, INFLAMMATORY_RESPONSE, and IL6_JAK_STAT3_SIGNALING gene sets (Figure 1G). GO enrichment analysis revealed enrichment of inflammation-related biological processes and vesicle-related cellular components (Figure S2B–D).

Heatmap analysis showed the upregulation of representative inflammation-related genes (Figure 1H). RT-qPCR further confirmed the upregulated expression of selected genes involved in inflammatory signaling, including TLR2, RELB, IL6, PTGS2, and TNFAIP3, following *F. nucleatum* stimulation (Figure 1I). In addition to the inflammatory transcriptional changes described above, heatmap analysis also revealed the upregulation of genes involved in vesicle trafficking, including PLD1, UGCG, MYO1B, STX11, PIGR, and RAB3IL1, following *F. nucleatum* stimulation (Figure 1H, Figure S2A). Consistently, GO enrichment analysis showed significant enrichment of vesicle- and membrane-related cellular component terms (Figure S2B–D). Together, these findings indicate that *F. nucleatum* stimulation is associated with inflammatory signaling and transcriptional alterations in vesicle trafficking-related genes.

### 3.2. F. nucleatum Stimulation Increases Exosome Yield from OSCC Cells

**Figure 2:**
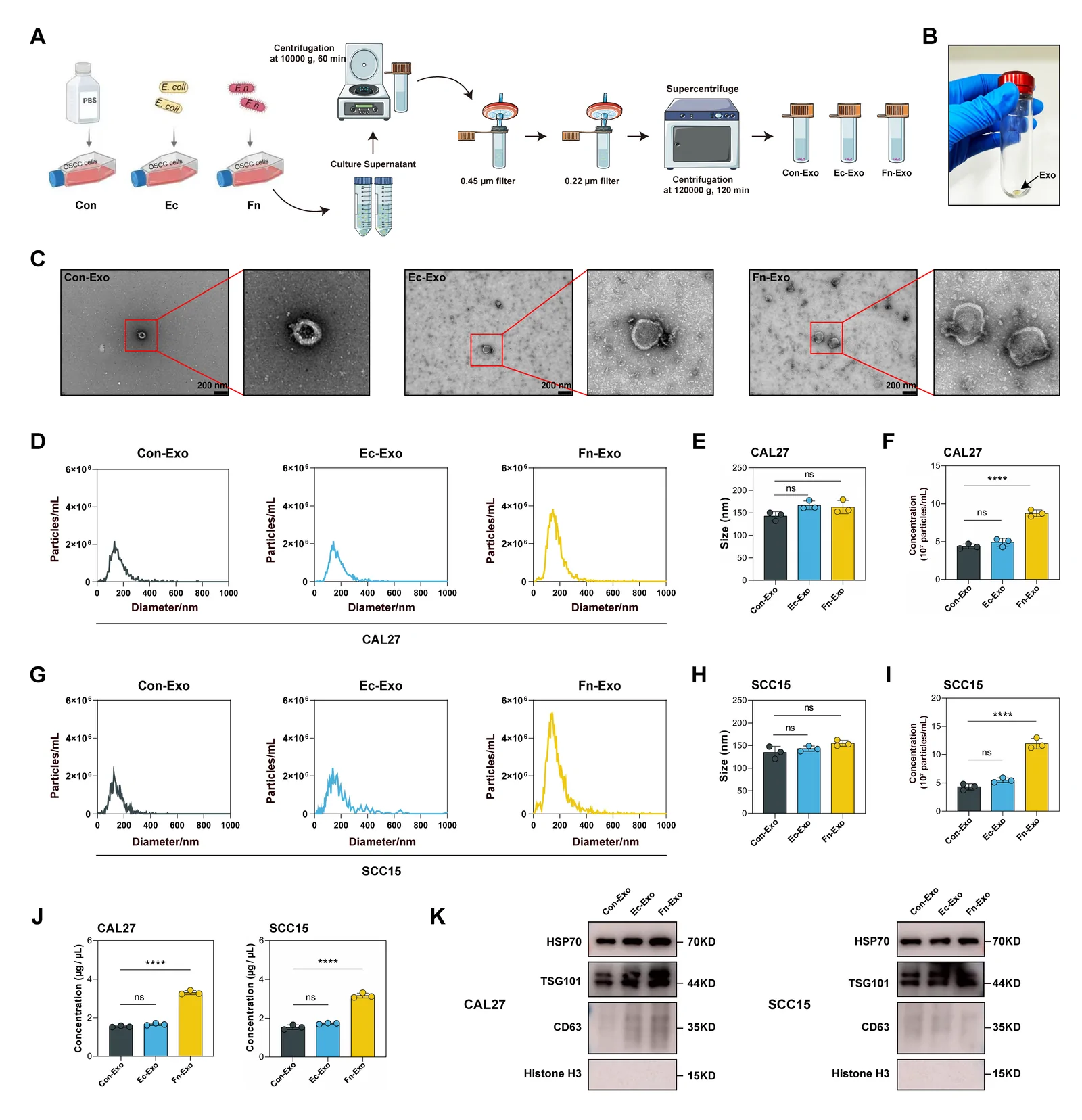
*F. nucleatum* stimulation increases exosome yield from OSCC cells. (**A**) Schematic illustration of extracellular vesicle isolation from OSCC cells treated with PBS, *E. coli*, or *F. nucleatum*. (**B**) Representative images of extracellular vesicle pellets obtained by differential ultracentrifugation. (**C**) Representative TEM images of purified Con-Exo, Ec-Exo, and Fn-Exo. Scale bar, 200 nm. (**D**–**I**) NTA analysis of extracellular vesicles isolated from CAL27 and SCC15 cells. Representative particle size distribution profiles are shown in (**D**,**G**). Mean particle size (**E**,**H**) and particle concentration (**F**,**I**) were quantified (*n* = 3). (**J**) Total protein concentrations of equal-volume Con-Exo, Ec-Exo, and Fn-Exo preparations from CAL27 and SCC15 cells, as determined by BCA assay (*n* = 3). (**K**) Western blot analysis of the exosome markers HSP70, CD63, and TSG101 and the negative marker histone H3. Data are presented as mean ± SD. Statistical significance was determined by one-way ANOVA followed by Dunnett’s multiple-comparisons test in (**E**,**F**,**H**,**I**,**J**). ns, not significant; **** *p* < 0.0001.

To examine whether *F. nucleatum* stimulation affects exosomes derived from OSCC cells, exosomes were isolated by differential ultracentrifugation from the conditioned media of OSCC cells treated with PBS, *E. coli*, or *F. nucleatum* and designated Con-Exo, Ec-Exo, and Fn-Exo, respectively (Figure 2A,B). Transmission electron microscopy (TEM) showed that Con-Exo, Ec-Exo, and Fn-Exo exhibited typical cup-shaped morphology (Figure 2C). Nanoparticle tracking analysis (NTA) (Figure 2D,G) showed no significant difference in mean particle size among Con-Exo, Ec-Exo, and Fn-Exo (Figure 2E,H). However, the particle concentration was significantly higher in Fn-Exo (8.57 ± 0.23 × 10^7^ particles/mL) than in Con-Exo (4.47 ± 0.21 × 10^7^ particles/mL) and Ec-Exo (4.63 ± 0.31 × 10^7^ particles/mL) (Figure 2F,I). After normalization to the corresponding cell number at the end of the exosome collection period, the particle yield remained significantly higher in the *F. nucleatum*-stimulated group (Figure S4). Moreover, BCA assay showed that equal-volume Fn-Exo preparations contained significantly higher amounts of total protein than Con-Exo and Ec-Exo preparations (Figure 2J). The particle-to-protein ratio was comparable among the CAL27-derived preparations, whereas SCC15-derived Fn-Exo showed a modest but significant increase compared with Con-Exo (Figure S5). Western blot analysis showed that Con-Exo, Ec-Exo, and Fn-Exo were positive for HSP70, TSG101, and CD63 but negative for histone H3, further supporting their characterization as exosomes (Figure 2K). Collectively, these results show that *F. nucleatum* stimulation increases the yield of exosomes recovered from OSCC cell-conditioned media.

### 3.3. Fn-Exo Are Internalized by LECs and Enhance the Pro-Adhesive Phenotype of LECs

**Figure 3:**
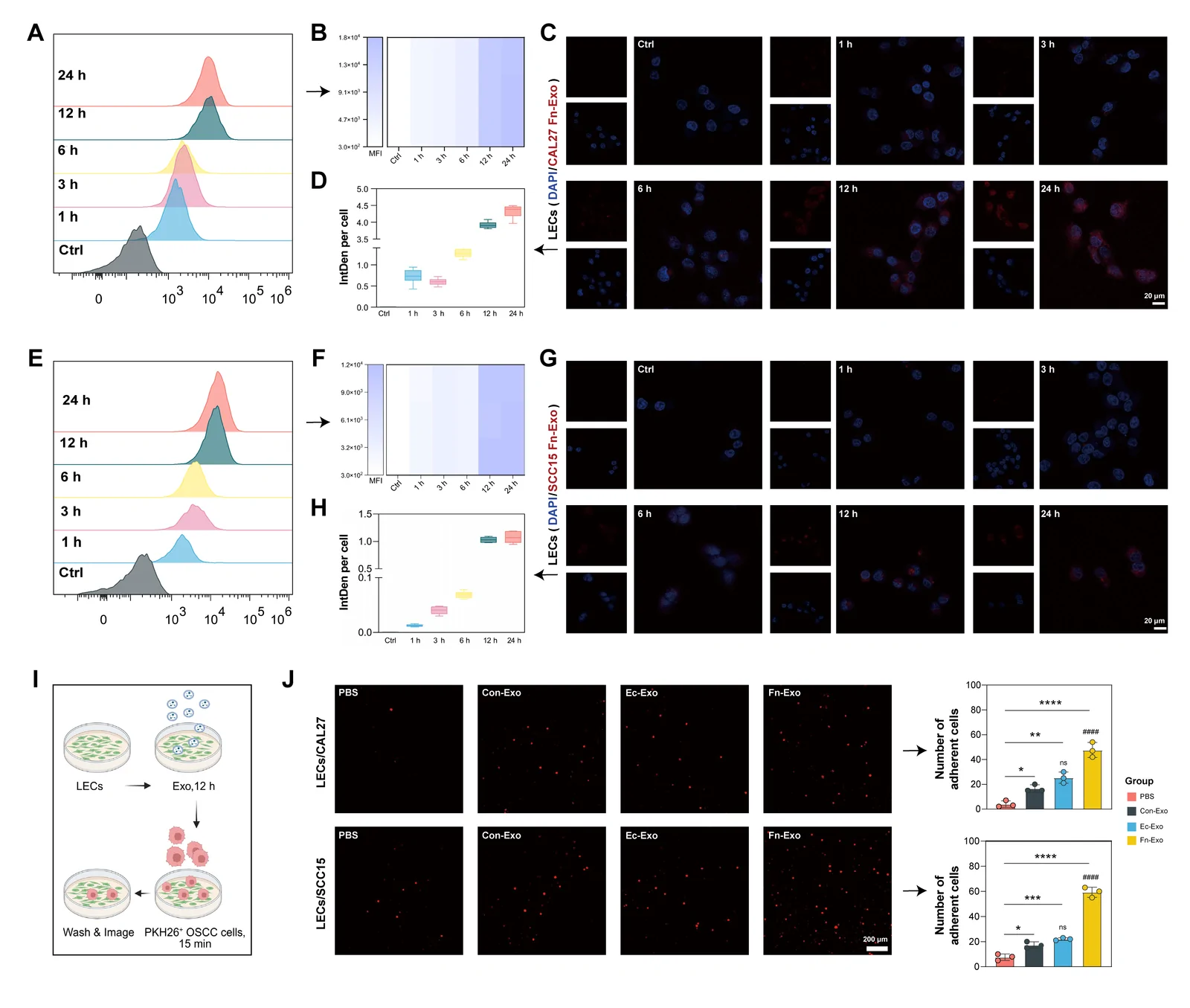
Fn-Exo are internalized by LECs and promote a pro-adhesive phenotype in LECs. (**A**–**H**) PKH26-labeled exosomes derived from *F. nucleatum*-treated CAL27 cells (**A**–**D**) or SCC15 cells (**E**–**H**) were incubated with LECs for 1, 3, 6, 12, or 24 h. Exosome uptake was evaluated by flow cytometry and confocal microscopy. Representative flow cytometry plots, fluorescence images, and quantitative analyses are shown (*n* = 3). Scale bar, 20 μm. (**I**) Schematic illustration of the in vitro adhesion assay. (**J**) CAL27 or SCC15 cells were subjected to adhesion assays after the indicated pretreatment of LECs. Representative fluorescence images and quantification of adherent tumor cells are shown (*n* = 3). Scale bar, 200 μm. Data are presented as mean ± SD. Statistical significance was determined by one-way ANOVA followed by Tukey’s multiple-comparisons test in (**J**). *, comparison with the PBS group; #, comparison with the Con-Exo group. ns, not significant; * *p* < 0.05, ** *p* < 0.01, *** *p* < 0.001, ****/#### *p* < 0.0001.

We first examined whether Fn-Exo could be internalized by LECs. To do this, 10 μg PKH26-labeled Fn-Exo were incubated with 1 × 10^5^ LECs for 1, 3, 6, 12, or 24 h. Flow cytometry showed a time-dependent increase in Fn-Exo-positive LECs, with uptake approaching a plateau at 12 h (Figure 3A,B,E,F). Minimal fluorescence was observed in the dye-only control (Figure S6). Confocal microscopy and fluorescence quantification showed a similar uptake pattern (Figure 3C,D,G,H). A 12 h pretreatment was therefore used in subsequent assays. We next evaluated whether Fn-Exo altered the adhesive phenotype of LECs. LEC monolayers were pretreated for 12 h with equal amounts of Con-Exo, Ec-Exo, or Fn-Exo, followed by incubation with PKH26-labeled CAL27 or SCC15 cells for 15 min (Figure 3I). As Figure 3J shows, compared with Con-Exo, Fn-Exo significantly enhanced tumor cell adhesion to LEC monolayers in both the CAL27 and SCC15 models. Con-Exo also increased adhesion relative to untreated controls. In contrast, Ec-Exo did not further increase adhesion compared with Con-Exo. No significant changes in LECs viability, morphology, or permeability were observed following Fn-Exo treatment compared with Con-Exo treatment (Figures S7–S9). Together, these results show that exosomes derived from *F. nucleatum*-stimulated OSCC cells induce a stronger pro-adhesive phenotype in LECs than control exosomes.

### 3.4. ICAM1 Contributes to the Adhesive Phenotype of LECs Induced by Fn-Exo

**Figure 4:**
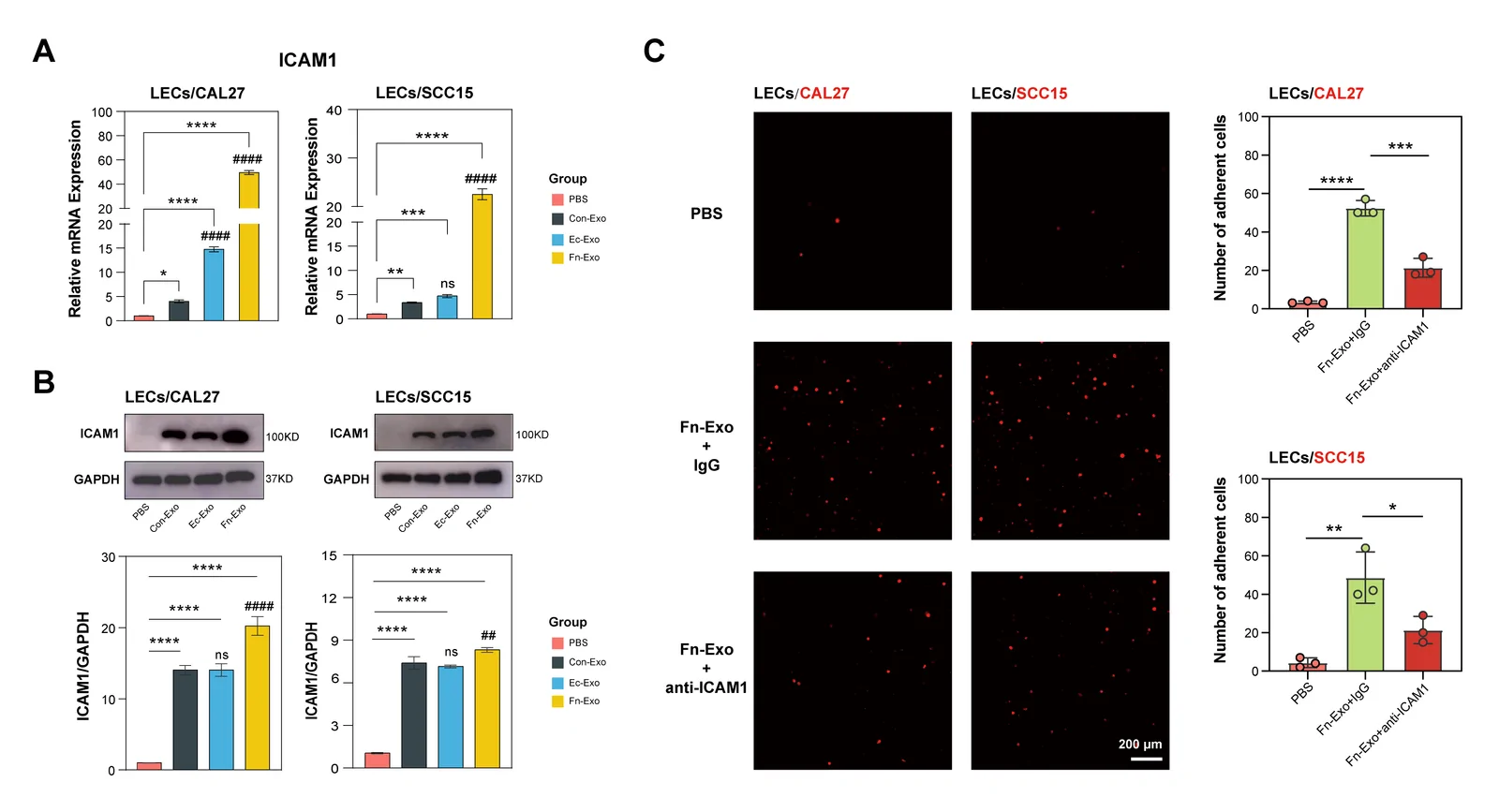
ICAM1 contributes to the pro-adhesive phenotype of LECs induced by Fn-Exo. (**A**) RT-qPCR analysis of ICAM1 mRNA expression in LECs treated with PBS, Con-Exo, Ec-Exo, or Fn-Exo (*n* = 3). (**B**) Western blot analysis of ICAM1 protein expression in LECs treated with the indicated exosomes and quantification of relative protein levels (*n* = 3). (**C**) LECs pretreated with Fn-Exo were incubated with IgG or anti-ICAM1 antibody and subjected to adhesion assays. Representative images and quantification of adherent OSCC cells are shown (*n* = 3). Scale bar, 200 μm. Data are presented as mean ± SD. Statistical significance was determined by one-way ANOVA followed by Tukey’s multiple-comparisons test in (**A**–**C**). #, Comparison with Con-Exo. ns, not significant; * *p* < 0.05, ##/** *p* < 0.01, *** *p* < 0.001, ####/**** *p* < 0.0001.

We next examined whether Fn-Exo altered ICAM1 expression in LECs. RT-qPCR and western blot analyses showed that Fn-Exo increased ICAM1 expression at both the mRNA and protein levels (Figure 4A,B). Fn-Exo treatment also resulted in a modest increase in VCAM1 expression (Figure S10). To assess the functional contribution of ICAM1, LECs were treated with an ICAM1-blocking antibody before the adhesion assay. ICAM1 blockade partially reduced the enhanced adhesion of OSCC cells to Fn-Exo-treated LECs (Figure 4C). These data support that ICAM1 contributes to the pro-adhesive phenotype induced by Fn-Exo in LECs.

### 3.5. Fn-Exo Promote ICAM1 Expression and a Pro-Adhesive Phenotype in LECs Through ROS/NF-κB Signaling

**Figure 5:**
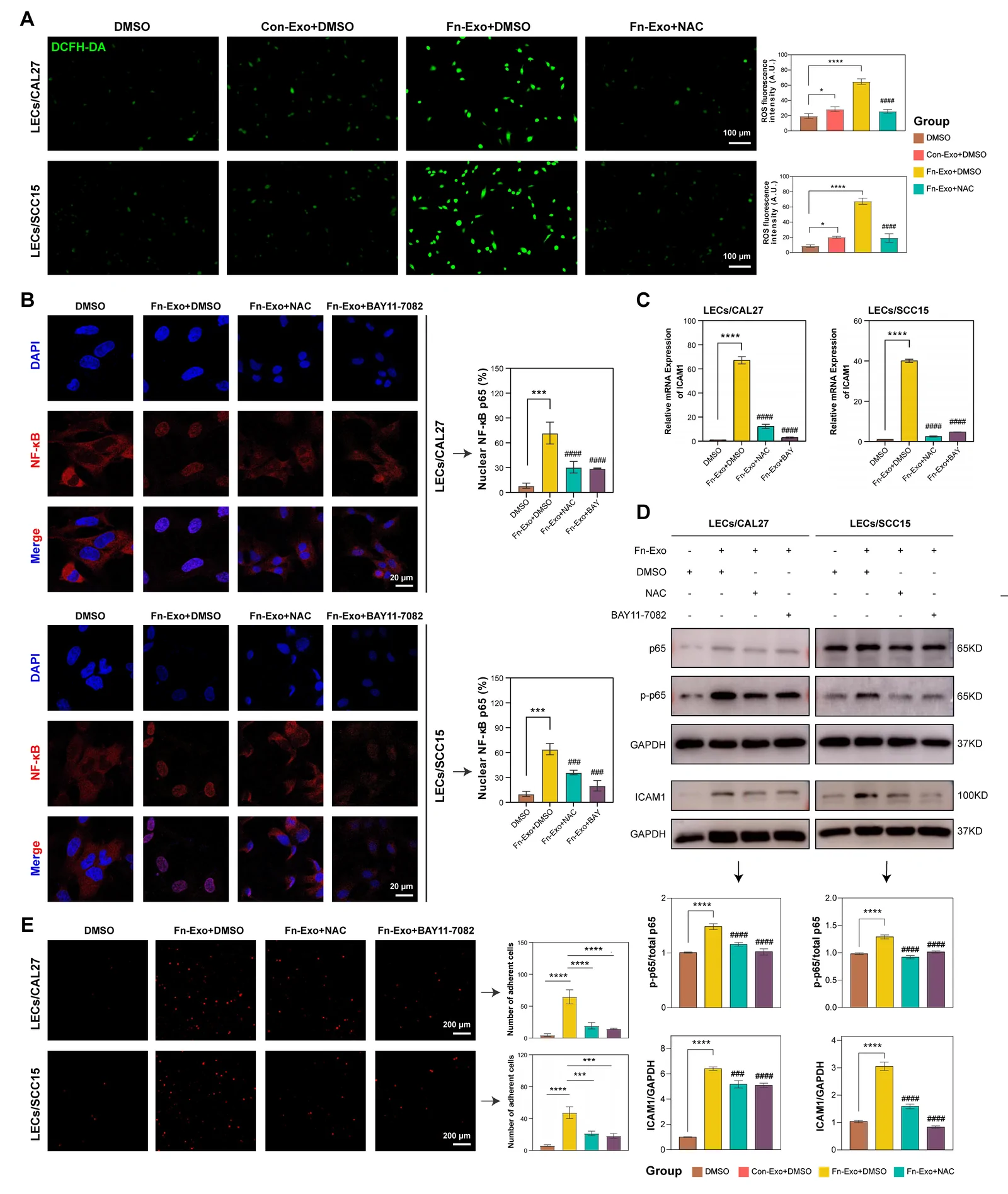
Fn-Exo induce ICAM1 expression and a pro-adhesive phenotype in LECs through ROS/NF-κB signaling. (**A**) LECs were treated with DMSO, Con-Exo + DMSO, Fn-Exo + DMSO, or Fn-Exo + NAC, and intracellular ROS levels were detected by DCFH-DA staining. Representative fluorescence images and quantification of ROS fluorescence intensity are shown (*n* = 3). Scale bar, 100 μm. (**B**) NF-κB p65 nuclear translocation was evaluated by immunofluorescence. Representative images were shown, and the nuclear-to-cytoplasmic fluorescence intensity ratio of p65 was quantified (*n* = 3). Scale bar, 20 μm. (**C**) RT-qPCR analysis of ICAM1 mRNA expression in LECs treated with DMSO, Fn-Exo + DMSO, Fn-Exo + NAC, or Fn-Exo + BAY11-7082 (*n* = 3). (**D**) Western blot analysis of phospho-p65, p65, ICAM1, and GAPDH was performed, and relative protein levels were quantified (*n* = 3). (**E**) LECs with the indicated treatments were subjected to an adhesion assay. Representative images and quantification of adherent CAL27 or SCC15 cells are shown (*n* = 3). Scale bar, 200 μm. Data are shown as mean ± SD. Statistical significance was determined by one-way ANOVA followed by Sidak’s multiple-comparisons test in (**A**–**E**). #, comparison with the Con-Exo + DMSO group in (**A**), comparison with the Fn-Exo + DMSO group in (**B**–**E**). ns, not significant; * *p* < 0.05, ###/*** *p* < 0.001, ####/**** *p* < 0.0001.

We next investigated the signaling mechanisms underlying Fn-Exo-induced ICAM1 expression. Fn-Exo markedly increased intracellular DCF fluorescence in LECs, whereas this effect was largely attenuated by NAC treatment (Figure 5A). We subsequently examined whether ROS accumulation was associated with NF-κB activation and ICAM1 expression. Fn-Exo promoted p65 nuclear translocation and increased the phospho-p65/p65 ratio, and both responses were attenuated by NAC or the NF-κB inhibitor BAY 11-7082 (Figure 5B,D). Notably, either NAC treatment or NF-κB inhibition also reduced Fn-Exo-induced ICAM1 expression at both the mRNA and protein levels (Figure 5C,D). These findings indicated that ROS accumulation contributes to NF-κB activation and subsequent ICAM1 upregulation. Functionally, adhesion assays showed that NAC and BAY 11-7082 each partially reduced the enhanced adhesion of CAL27 and SCC15 cells to Fn-Exo-treated LEC monolayers (Figure 5E). Taken together, these findings support the involvement of ROS/NF-κB signaling in Fn-Exo-induced ICAM1 upregulation and the resulting pro-adhesive phenotype of LECs.

## 4. Discussion

In this study, we show that *F. nucleatum* stimulation of OSCC cells elicits a functional response in LECs through exosomes. The involvement of exosomes is particularly relevant because lymphatic vessels and draining lymph nodes are highly exposed to extracellular vesicles released from tumors and stromal cells [12,21,30,31,32,33,34]. Previous studies have shown that tumor-derived or tissue-derived exosomes can be taken up by endothelial cells and modify endothelial proliferation, migration, tube formation, immune-regulatory activity, and lymphatic remodeling [35,36,37,38]. In OSCC, hypoxic tumor-derived exosomes were reported to promote lymphangiogenesis by altering the miR-128-3p/VEGF-C axis [16], while studies in other tumor models have shown that exosomal CXCR4, LEC-derived exosomal miRNAs, ADSC-derived exosomal miR-132, hypoxia-conditioned ADSC-derived vesicles, and exosomal miR-10527-5p can regulate LEC behavior and lymphatic metastasis-related phenotypes [39,40,41,42,43]. Fn-Exo induced a pro-adhesive phenotype in LECs, which may contribute to tumor–lymphatic endothelial interactions associated with lymphatic dissemination. These findings extend previous studies by identifying tumor-derived exosomes as mediators linking *F. nucleatum*-stimulated OSCC cells to a pro-adhesive phenotype in LECs.

ICAM1 is a classical inducible adhesion molecule in endothelial inflammation and is also involved in cancer cell–endothelium interactions [24,44]. In LECs, inflammatory stimuli can induce ICAM1 and VCAM1 expression, thereby altering leukocyte and dendritic cell trafficking through lymphatic vessels [45,46]. In tumor backgrounds, CCL2-induced ICAM1 facilitates cancer cell–LEC interactions in sentinel lymph nodes [24], melanoma-derived small extracellular vesicles induce ICAM1 in LECs to support tumor cell adhesion [21], and ICAM1 has also been implicated in lymphatic barrier disruption and cancer cell transmigration [47,48]. The partial reduction of adhesion following ICAM1 blockade supports a functional contribution of ICAM1 to the Fn-Exo-induced pro-adhesive phenotype, while suggesting that additional adhesion molecules or other adhesion-related mechanisms may also contribute. These interpretations are consistent with the broader role of endothelial ICAM1 in tumor cell clustering, heterotypic tumor–endothelium adhesion, and transendothelial migration [49].

ROS can function as intracellular second messengers and promote NF-κB activation under inflammatory conditions [42]. Skeletal muscle-derived exosomes were shown to activate endothelial functions through ROS-dependent NF-κB signaling [35], and multiple endothelial models have demonstrated that ROS generation can induce ICAM1 expression and leukocyte or monocyte adhesion [50,51]. In addition, visfatin-induced ICAM1 and VCAM1 expression in endothelial cells depends on ROS-mediated NF-κB activation [52], and extracellular histones can trigger a TLR4/ROS/NF-κB/cell adhesion molecule pathway in endothelial cells [53]. Our findings support a model in which Fn-Exo induce ROS accumulation, thereby promoting NF-κB activation and subsequent ICAM1 upregulation. Moreover, the partial reversal of the adhesive phenotype by NAC and BAY11-7082 suggested that ROS/NF-κB/ICAM1 represents one important pathway involved in the Fn-Exo-induced pro-adhesive phenotype.

Our findings suggest that *F. nucleatum* may contribute to remodeling of the lymphatic endothelial interface through exosome-mediated communication. Clinically, the enhanced adhesion of OSCC cells to Fn-Exo-treated LECs may favor tumor cell attachment to lymphatic endothelium, a process that is relevant to cervical lymph node spread. However, the present work is based mainly on in vitro OSCC-LEC models, and the specific *F. nucleatum*-derived components responsible for modulating exosome yield, as well as the exosomal cargo mediating the downstream effects, remain unidentified. Future studies should include complementary genetic validation of the signaling pathway, examine whether Fn-Exo accumulate in draining lymph nodes in vivo, identify the specific exosomal cargo responsible for ROS induction, determine whether additional adhesion molecules cooperate with ICAM1, and examine whether the abundance of *F. nucleatum*, Fn-Exo signatures, and ICAM1-positive LECs correlate with cervical lymph node metastasis in OSCC patient cohorts.

## 5. Conclusions

In summary, *F. nucleatum* was detected in OSCC tissues and induced inflammation- and vesicle-associated transcriptional changes in OSCC cells. *F. nucleatum* stimulation increased the yield of recovered exosomes, and the resulting Fn-Exo were internalized by LECs and enhanced OSCC cell adhesion to LEC monolayers. Fn-Exo treatment was associated with ROS accumulation, NF-κB activation, and ICAM1 upregulation in LECs. ROS scavenging, NF-κB inhibition, and ICAM1 blockade partially attenuated the enhanced adhesive phenotype. Together, these findings support the involvement of ROS/NF-κB/ICAM1 signaling in the Fn-Exo-induced pro-adhesive phenotype in LECs.

## Data Availability

The RNA-seq data have been deposited in the NCBI SRA under BioProject accession number PRJNA1496439. The original contributions presented in the study are included in the article/Supplementary Materials, and further inquiries can be directed to the corresponding authors.

## Supplementary Materials

The following supporting information can be downloaded at https://www.mdpi.com/article/10.3390/microorganisms14091965/s1, Figure S1. Correlation patterns among bacterial genera identified from OSCC transcriptomic datasets. (A) Pairwise correlation heatmap of bacterial genera detected in 123 OSCC samples from the GSE184616 (*n* = 15) and GSE275870 (*n* = 108) datasets. Hierarchical clustering was performed based on the correlation matrix. Red and blue indicate positive and negative correlations, respectively, with color intensity representing the correlation strength. Dashed boxes denote the major correlation clusters, and *Fusobacterium* is highlighted in red. Figure S2. Heatmap and Gene Ontology (GO) enrichment analyses of transcriptional changes induced by *F. nucleatum* in OSCC cells. (A) Heatmap of differentially expressed genes in control and *F. nucleatum*-stimulated OSCC cells. Selected genes are indicated on the right. (B–D) GO enrichment analysis of all DEGs (All), upregulated genes (UP), and downregulated genes (DOWN), categorized into biological process (BP; B), cellular component (CC; C), and molecular function (MF; D). Bubble size represents the gene ratio, and bubble color indicates the enrichment *p* value. Figure S3. CAL27 and SCC15 cell counts following *F. nucleatum* stimulation. Cell numbers were determined at 0, 24, and 48 h. Data are presented as mean ± SD (*n* = 3). Statistical significance was assessed by two-way ANOVA. ns, not significant; *** *p* < 0.001; **** *p* < 0.0001. Figure S4. Cell-normalized exosome particle yield from CAL27 and SCC15 cells following *F. nucleatum* stimulation. Exosome particle numbers measured by NTA were normalized to the corresponding cell numbers determined at the end of the exosome collection period. Data are presented as mean ± SD (*n* = 3). Statistical significance was assessed by one-way ANOVA. ns, not significant; ** *p* < 0.01. Figure S5. The particle-to-protein ratio (particles/μg protein) was calculated from the particle concentration measured by NTA, and the total protein concentration was determined by BCA assay. Data are presented as mean ± SD. Statistical significance was determined by one-way ANOVA test. ns, not significant; * *p* < 0.05. Figure S6. Representative fluorescence image of LECs treated with PKH26 in the absence of exosomes. Scale bar, 20 μm. Figure S7. LEC viability following treatment with PBS, Con-Exo, or Fn-Exo, as assessed by CCK-8 assay. Data are presented as mean ± SD from three independent biological replicates. Statistical significance was determined by one-way ANOVA. ns, not significant. Figure S8. Morphological assessment of LECs following exosome treatment. Representative fluorescence images of LECs treated with PBS, Con-Exo, or Fn-Exo derived from CAL27 or SCC15 cells. F-actin was stained with phalloidin (red), and nuclei were counterstained with DAPI (blue). Scale bar, 20 μm. Figure S9. Endothelial permeability following exosome treatment. Relative permeability of LEC monolayers treated with CAL27 or SCC15 derived Con-Exo and Fn-Exo was assessed by FITC-dextran flux assay. Statistical significance was assessed by two-tailed unpaired Student’s *t*-test. Data are shown as mean ± SD (*n* = 3). ns, not significant. Figure S10. Expression of ICAM1 and VCAM1 in LECs following treatment with OSCC-derived exosomes. Relative mRNA expression of ICAM1 and VCAM1 in LECs treated with Con-Exo or Fn-Exo derived from CAL27 or SCC15 cells. Data are shown as mean ± SD (*n* = 3). Statistical significance was assessed by two-tailed unpaired Student’s *t*-test. ** *p* < 0.01, *** *p* < 0.001, ** *p* < 0.0001. Table S1. Primer sequences used for RT-qPCR. Table S2. Differentially expressed genes between *F. nucleatum*-treated and control OSCC cells.

## References

[1] Tan Y., Wang Z., Xu M., Li B., Huang Z., Qin S., Nice E.C., Tang J., Huang C. (2023). Oral squamous cell carcinomas: State of the field and emerging directions. Int. J. Oral Sci..

[2] Yu Y.F., Cao L.M., Li Z.Z., Zhong N.N., Wang G.R., Xiao Y., Wu Q.J., Liu B., Bu L.L. (2025). Frequency of lymph node metastases at different neck levels in patients with oral squamous cell carcinoma: A systematic review and meta-analysis. Int. J. Surg..

[3] Johnson D.E., Burtness B., Leemans C.R., Lui V.W.Y., Bauman J.E., Grandis J.R. (2020). Head and neck squamous cell carcinoma. Nat. Rev. Dis. Primers.

[4] McIlvanna E., Linden G.J., Craig S.G., Lundy F.T., James J.A. (2021). Fusobacterium nucleatum and oral cancer: A critical review. BMC Cancer.

[5] Chiscuzzu F., Crescio C., Varrucciu S., Rizzo D., Sali M., Delogu G., Bussu F. (2025). Current Evidence on the Relation Between Microbiota and Oral Cancer-The Role of Fusobacterium nucleatum-A Narrative Review. Cancers.

[6] Rubinstein M.R., Wang X., Liu W., Hao Y., Cai G., Han Y.W. (2013). Fusobacterium nucleatum promotes colorectal carcinogenesis by modulating E-cadherin/β-catenin signaling via its FadA adhesin. Cell Host Microbe.

[7] Kostic A.D., Chun E., Robertson L., Glickman J.N., Gallini C.A., Michaud M., Clancy T.E., Chung D.C., Lochhead P., Hold G.L. (2013). Fusobacterium nucleatum potentiates intestinal tumorigenesis and modulates the tumor-immune microenvironment. Cell Host Microbe.

[8] Yang Y.Z., Weng W.H., Peng J.J., Hong L.M., Yang L., Toiyama Y., Gao R.Y., Liu M.F., Yin M.M., Pan C. (2017). Fusobacterium nucleatum Increases Proliferation of Colorectal Cancer Cells and Tumor Development in Mice by Activating Toll-Like Receptor 4 Signaling to Nuclear Factor-κB, and Up-regulating Expression of MicroRNA-21. Gastroenterology.

[9] Sun J., Tang Q., Yu S., Xie M., Zheng W., Chen G., Yin Y., Huang X., Wo K., Lei H. (2023). F. nucleatum facilitates oral squamous cell carcinoma progression via GLUT1-driven lactate production. EBioMedicine.

[10] Bullman S., Pedamallu C.S., Sicinska E., Clancy T.E., Zhang X., Cai D., Neuberg D., Huang K., Guevara F., Nelson T. (2017). Analysis of Fusobacterium persistence and antibiotic response in colorectal cancer. Science.

[11] Kalluri R., LeBleu V.S. (2020). The biology, function, and biomedical applications of exosomes. Science.

[12] Becker A., Thakur B.K., Weiss J.M., Kim H.S., Peinado H., Lyden D. (2016). Extracellular Vesicles in Cancer: Cell-to-Cell Mediators of Metastasis. Cancer Cell.

[13] Gao J., Zhang X., Jiang L., Li Y., Zheng Q. (2022). Tumor endothelial cell-derived extracellular vesicles contribute to tumor microenvironment remodeling. Cell Commun. Signal..

[14] Guo S.H., Chen J., Chen F.F., Zeng Q.Y., Liu W.L., Zhang G. (2021). Exosomes derived from Fusobacterium nucleatum-infected colorectal cancer cells facilitate tumour metastasis by selectively carrying miR-1246/92b-3p/27a-3p and CXCL16. Gut.

[15] Hui B., Zhou C., Xu Y., Wang R., Dong Y., Zhou Y., Ding J., Zhang X., Xu J., Gu Y. (2024). Exosomes secreted by Fusobacterium nucleatum-infected colon cancer cells transmit resistance to oxaliplatin and 5-FU by delivering hsa_circ_0004085. J. Nanobiotechnol..

[16] You T., Mao F., Zhang K., Zhang X., Hu X. (2026). Hypoxia-Mediated Downregulation of Exosomal miR-128-3p Promotes Lymphangiogenesis via VEGF-C in Oral Squamous Cell Carcinoma. Int. J. Dent..

[17] Capik O., Gumus R., Karatas O.F. (2023). Hypoxia-induced tumor exosomes promote angiogenesis through miR-1825/TSC2/mTOR axis in oral squamous cell carcinoma. Head Neck.

[18] Alitalo A., Detmar M. (2012). Interaction of tumor cells and lymphatic vessels in cancer progression. Oncogene.

[19] Karaman S., Detmar M. (2014). Mechanisms of lymphatic metastasis. J. Clin. Invest..

[20] Hirakawa S., Kodama S., Kunstfeld R., Kajiya K., Brown L.F., Detmar M. (2005). VEGF-A induces tumor and sentinel lymph node lymphangiogenesis and promotes lymphatic metastasis. J. Exp. Med..

[21] García-Silva S., Benito-Martín A., Nogués L., Hernández-Barranco A., Mazariegos M.S., Santos V., Hergueta-Redondo M., Ximénez-Embún P., Kataru R.P., Lopez A.A. (2021). Melanoma-derived small extracellular vesicles induce lymphangiogenesis and metastasis through an NGFR-dependent mechanism. Nat. Cancer.

[22] Hood J.L., San R.S., Wickline S.A. (2011). Exosomes released by melanoma cells prepare sentinel lymph nodes for tumor metastasis. Cancer Res..

[23] Mejía-Rangel J., Córdova E., Orozco L., Ventura-Gallegos J.L., Mitre-Aguilar I., Escalona-Guzmán A., Vadillo F., Vázquez-Prado J., Gariglio P., Zentella-Dehesa A. (2016). Pro-adhesive phenotype of normal endothelial cells responding to metastatic breast cancer cell conditioned medium is linked to NFκB-mediated transcriptomic regulation. Int. J. Oncol..

[24] Kawai Y., Kaidoh M., Yokoyama Y., Sano K., Ohhashi T. (2009). Chemokine CCL2 facilitates ICAM-1-mediated interactions of cancer cells and lymphatic endothelial cells in sentinel lymph nodes. Cancer Sci..

[25] Wei S., Wu X., Chen M., Xiang Z., Li X., Zhang J., Dong W. (2023). Exosomal-miR-129-2-3p derived from Fusobacterium nucleatum-infected intestinal epithelial cells promotes experimental colitis through regulating TIMELESS-mediated cellular senescence pathway. Gut Microbes.

[26] Au Yeung C.L., Co N.N., Tsuruga T., Yeung T.L., Kwan S.Y., Leung C.S., Li Y., Lu E.S., Kwan K., Wong K.K. (2016). Exosomal transfer of stroma-derived miR21 confers paclitaxel resistance in ovarian cancer cells through targeting APAF1. Nat. Commun..

[27] Webber J., Clayton A. (2013). How pure are your vesicles?. J. Extracell. Vesicles.

[28] Yang H., Bai Y., Fu C., Liu W., Diao Z. (2023). Exosomes from high glucose-treated macrophages promote epithelial-mesenchymal transition of renal tubular epithelial cells via long non-coding RNAs. BMC Nephrol..

[29] Zhang Y., Zhang L., Zheng S., Li M., Xu C., Jia D., Qi Y., Hou T., Wang L., Wang B. (2022). Fusobacterium nucleatum promotes colorectal cancer cells adhesion to endothelial cells and facilitates extravasation and metastasis by inducing ALPK1/NF-κB/ICAM1 axis. Gut Microbes.

[30] Théry C., Witwer K.W., Aikawa E., Alcaraz M.J., Anderson J.D., Andriantsitohaina R., Antoniou A., Arab T., Archer F., Atkin-Smith G.K. (2018). Minimal information for studies of extracellular vesicles 2018 (MISEV2018): A position statement of the International Society for Extracellular Vesicles and update of the MISEV2014 guidelines. J. Extracell. Vesicles.

[31] Peinado H., Alečković M., Lavotshkin S., Matei I., Costa-Silva B., Moreno-Bueno G., Hergueta-Redondo M., Williams C., García-Santos G., Ghajar C. (2012). Melanoma exosomes educate bone marrow progenitor cells toward a pro-metastatic phenotype through MET. Nat. Med..

[32] Hoshino A., Costa-Silva B., Shen T.L., Rodrigues G., Hashimoto A., Tesic Mark M., Molina H., Kohsaka S., Di Giannatale A., Ceder S. (2015). Tumour exosome integrins determine organotropic metastasis. Nature.

[33] Costa-Silva B., Aiello N.M., Ocean A.J., Singh S., Zhang H., Thakur B.K., Becker A., Hoshino A., Mark M.T., Molina H. (2015). Pancreatic cancer exosomes initiate pre-metastatic niche formation in the liver. Nat. Cell Biol..

[34] Guo Y., Ji X., Liu J., Fan D., Zhou Q., Chen C., Wang W., Wang G., Wang H., Yuan W. (2019). Effects of exosomes on pre-metastatic niche formation in tumors. Mol. Cancer.

[35] Nie Y., Sato Y., Garner R.T., Kargl C., Wang C., Kuang S., Gilpin C.J., Gavin T.P. (2019). Skeletal muscle-derived exosomes regulate endothelial cell functions via reactive oxygen species-activated nuclear factor-κB signalling. Exp. Physiol..

[36] Leary N., Walser S., He Y., Cousin N., Pereira P., Gallo A., Collado-Diaz V., Halin C., Garcia-Silva S., Peinado H. (2022). Melanoma-derived extracellular vesicles mediate lymphatic remodelling and impair tumour immunity in draining lymph nodes. J. Extracell. Vesicles.

[37] Zhou C., Wei W., Ma J., Yang Y., Liang L., Zhang Y., Wang Z., Chen X., Huang L., Wang W. (2021). Cancer-secreted exosomal miR-1468-5p promotes tumor immune escape via the immunosuppressive reprogramming of lymphatic vessels. Mol. Ther..

[38] Wang L., Li L., Zhu G. (2021). Role of Extracellular Vesicles on Cancer Lymphangiogenesis and Lymph Node Metastasis. Front. Oncol..

[39] Li M., Lu Y., Xu Y., Wang J., Zhang C., Du Y., Wang L., Li L., Wang B., Shen J. (2018). Horizontal transfer of exosomal CXCR4 promotes murine hepatocarcinoma cell migration, invasion and lymphangiogenesis. Gene.

[40] Kim K.S., Park J.I., Oh N., Cho H.J., Park J.H., Park K.S. (2019). ELK3 expressed in lymphatic endothelial cells promotes breast cancer progression and metastasis through exosomal miRNAs. Sci. Rep..

[41] Wang X., Wang H., Cao J., Ye C. (2018). Exosomes from Adipose-Derived Stem Cells Promotes VEGF-C-Dependent Lymphangiogenesis by Regulating miRNA-132/TGF-β Pathway. Cell. Physiol. Biochem..

[42] Yang Y., Li X.B., Li Y., Li T.X., Li P., Deng G.M., Guo Q., Zhou X., Chen X.H. (2022). Extracellular Vesicles Derived From Hypoxia-Conditioned Adipose-Derived Mesenchymal Stem Cells Enhance Lymphangiogenesis. Cell Transplant..

[43] Xiao Z., Feng X., Zhou Y., Li P., Luo J., Zhang W., Zhou J., Zhao J., Wang D., Wang Y. (2023). Exosomal miR-10527-5p Inhibits Migration, Invasion, Lymphangiogenesis and Lymphatic Metastasis by Affecting Wnt/β-Catenin Signaling via Rab10 in Esophageal Squamous Cell Carcinoma. Int. J. Nanomed..

[44] Haydinger C.D., Ashander L.M., Tan A.C.R., Smith J.R. (2023). Intercellular Adhesion Molecule 1: More than a Leukocyte Adhesion Molecule. Biology.

[45] Vigl B., Aebischer D., Nitschké M., Iolyeva M., Röthlin T., Antsiferova O., Halin C. (2011). Tissue inflammation modulates gene expression of lymphatic endothelial cells and dendritic cell migration in a stimulus-dependent manner. Blood.

[46] Johnson L.A., Clasper S., Holt A.P., Lalor P.F., Baban D., Jackson D.G. (2006). An inflammation-induced mechanism for leukocyte transmigration across lymphatic vessel endothelium. J. Exp. Med..

[47] Jin D., Otani K., Yamahara K., Ikeda T., Nagaya N., Kangawa K. (2011). Adrenomedullin reduces expression of adhesion molecules on lymphatic endothelial cells. Regul. Pept..

[48] Viola K., Kopf S., Huttary N., Vonach C., Kretschy N., Teichmann M., Giessrigl B., Raab I., Stary S., Krieger S. (2013). Bay11-7082 inhibits the disintegration of the lymphendothelial barrier triggered by MCF-7 breast cancer spheroids; the role of ICAM-1 and adhesion. Br. J. Cancer.

[49] Taftaf R., Liu X., Singh S., Jia Y., Dashzeveg N.K., Hoffmann A.D., El-Shennawy L., Ramos E.K., Adorno-Cruz V., Schuster E.J. (2021). ICAM1 initiates CTC cluster formation and trans-endothelial migration in lung metastasis of breast cancer. Nat. Commun..

[50] Chiu J.J., Wung B.S., Shyy J.Y., Hsieh H.J., Wang D.L. (1997). Reactive oxygen species are involved in shear stress-induced intercellular adhesion molecule-1 expression in endothelial cells. Arterioscler. Thromb. Vasc. Biol..

[51] Cheng J.J., Wung B.S., Chao Y.J., Wang D.L. (1998). Cyclic strain-induced reactive oxygen species involved in ICAM-1 gene induction in endothelial cells. Hypertension.

[52] Kim S.R., Bae Y.H., Bae S.K., Choi K.S., Yoon K.H., Koo T.H., Jang H.O., Yun I., Kim K.W., Kwon Y.G. (2008). Visfatin enhances ICAM-1 and VCAM-1 expression through ROS-dependent NF-kappaB activation in endothelial cells. Biochim. Biophys. Acta.

[53] Pérez-Cremades D., Bueno-Betí C., García-Giménez J.L., Ibañez-Cabellos J.S., Pallardó F.V., Hermenegildo C., Novella S. (2023). Extracellular histones trigger oxidative stress-dependent induction of the NF-kB/CAM pathway via TLR4 in endothelial cells. J. Physiol. Biochem..

