# Supplementary material for: Fusobacterium nucleatum-Stimulated OSCC Cell-Derived Exosomes Induce a Pro-Adhesive Phenotype in Lymphatic Endothelial Cells via the ROS/NF-κB/ICAM1 Axis
Source: Microorganisms. 2026 Sep 5;14(9):1965. doi: 10.3390/microorganisms14091965 (PMC13609351; doi:10.3390/microorganisms14091965)
Supplement: Supplementary file 1 [file microorganisms-14-01965-s001.zip › microorganisms-4476606-supplementary.pdf]

Supplementary Information

Fusobacterium nucleatum-Stimulated OSCC Cell-Derived Exosomes Induce a Pro-Adhesive Phenotype in Lymphatic Endothelial Cells *via* the ROS/NF-κB/ICAM1 Axis

Li Wei [1], Qi He [1], Haiting Gao [1], Wanheng Li [1], Tianyong Sun [1, 2]*, Qiang Feng [1, 2, 3, 4]*

[1] Department of Human Microbiome, School and Hospital of Stomatology, Cheeloo College of Medicine, Shandong University & Shandong Key Laboratory of Oral Diseases & Shandong Engineering Research Center of Dental Materials and Oral Tissue Regeneration & Shandong Provincial Clinical Research Center for Oral Diseases, Jinan 250012, China; 202221021@mail.sdu.edu.cn (L.W.)

[2] School of Stomatology, College of Life Sciences and Medicine, Northwest University, Xi'an 710069, China

[3] State Key Laboratory of Microbial Technology, Shandong University, Qingdao, China

[4] Shandong University-BOP Joint Oral Microbiome Laboratory, Jinan 250012, China

* Correspondence: sty@sdu.edu.cn (T.S.), fengqiang@sdu.edu.cn (Q.F.)

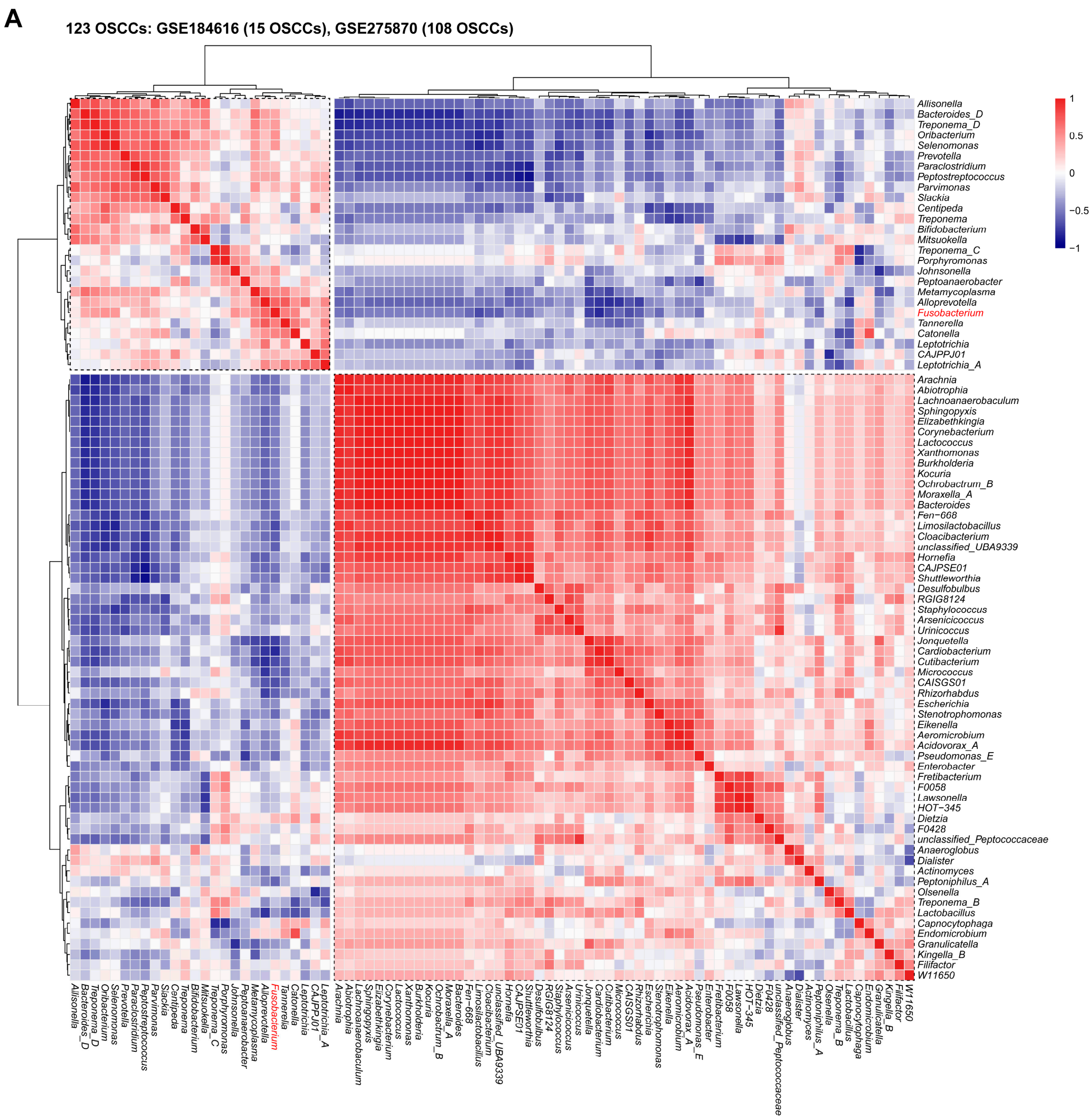

Figure S1. Correlation patterns among bacterial genera identified from OSCC transcriptomic datasets. (**A**) Pairwise correlation heatmap of bacterial genera detected in 123 OSCC samples from the GSE184616 (n = 15) and GSE275870 (n = 108) datasets. Hierarchical clustering was performed based on the correlation matrix. Red and blue indicate positive and negative correlations, respectively, with color intensity representing the correlation strength. Dashed boxes denote the major correlation clusters, and *Fusobacterium* is highlighted in red.

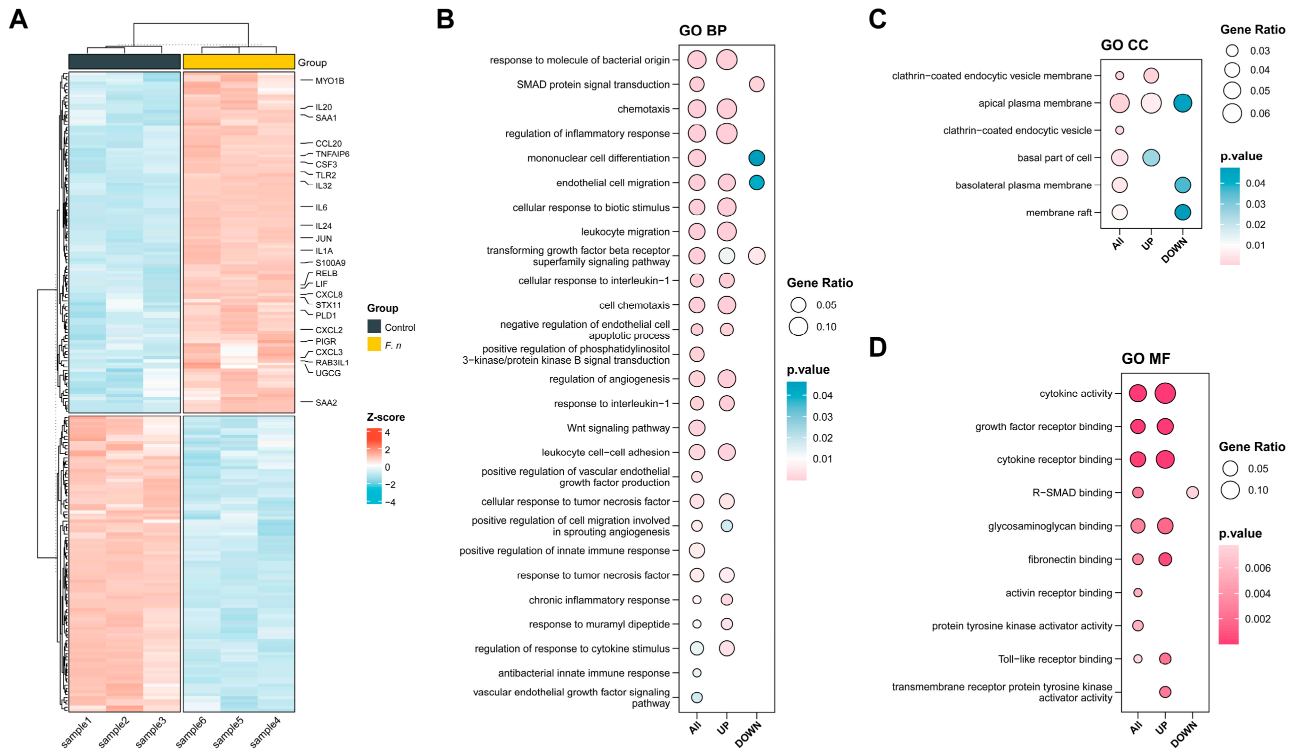

Figure S2. Heatmap and Gene Ontology (GO) enrichment analyses of transcriptional changes induced by *F. nucleatum* in OSCC cells. (**A**) Heatmap of differentially expressed genes in control and *F. nucleatum*-stimulated OSCC cells. Selected genes are indicated on the right. (**B–D**) GO enrichment analysis of all DEGs (All), upregulated genes (UP), and downregulated genes (DOWN), categorized into biological process (BP; B), cellular component (CC; C), and molecular function (MF; D). Bubble size represents the gene ratio, and bubble color indicates the enrichment *p* value.

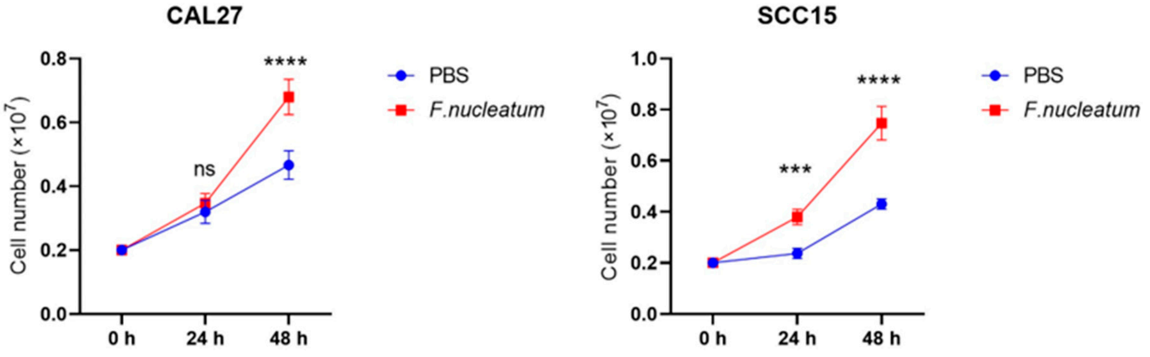

Figure S3. CAL27 and SCC15 cell counts following *F. nucleatum* stimulation. Cell numbers were determined at 0, 24, and 48 h. Data are presented as mean ± SD (n = 3). Statistical significance was assessed by two-way ANOVA. ns, not significant; ***$P < 0.001$; ****$P < 0.0001$.

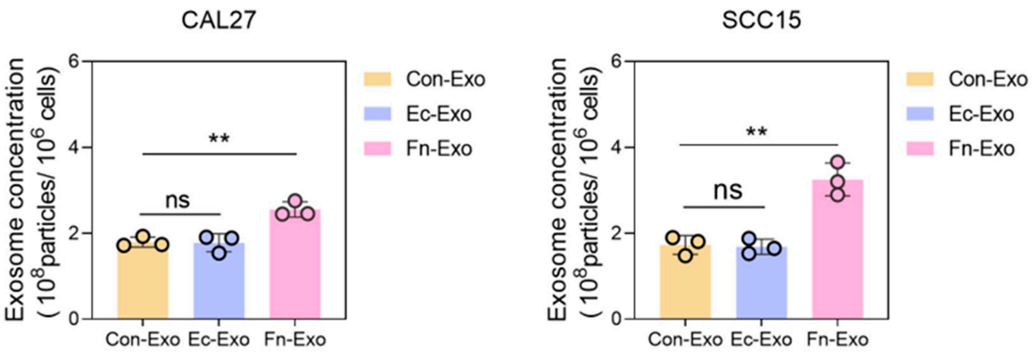

Figure S4. Cell-normalized exosome particle yield from CAL27 and SCC15 cells following *F. nucleatum* stimulation. Exosome particle numbers measured by NTA were normalized to the corresponding cell numbers determined at the end of the exosome collection period. Data are presented as mean ± SD (n = 3). Statistical significance was assessed by one-way ANOVA. ns, not significant; $^{**}P < 0.01$.

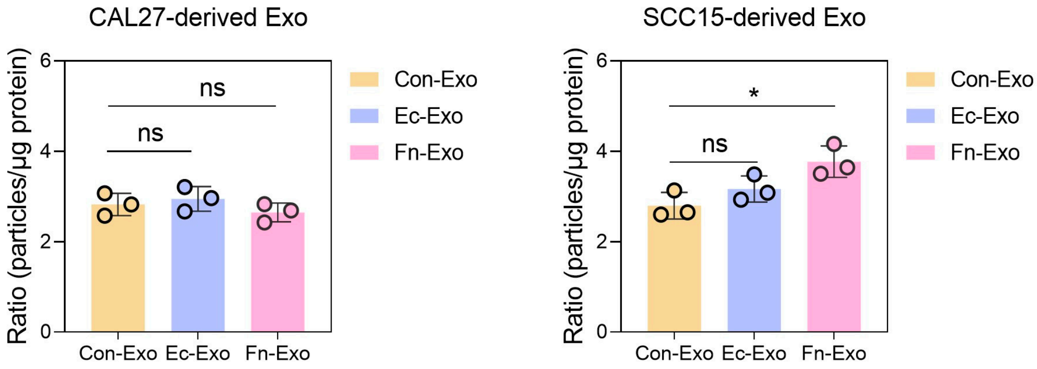

Figure S5. The particle-to-protein ratio (particles/μg protein) was calculated from the particle concentration measured by NTA and the total protein concentration determined by BCA assay. Data are presented as mean ± SD. Statistical significance was determined by one-way ANOVA test. ns, not significant; $^{*}P < 0.05$.

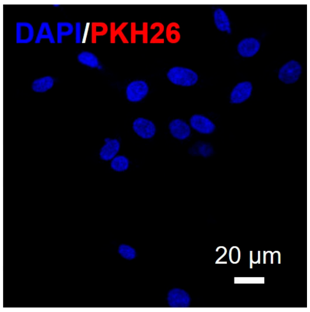

Figure S6. Representative fluorescence image of LECs treated with PKH26 in the absence of exosomes. Scale bar, 20 μm.

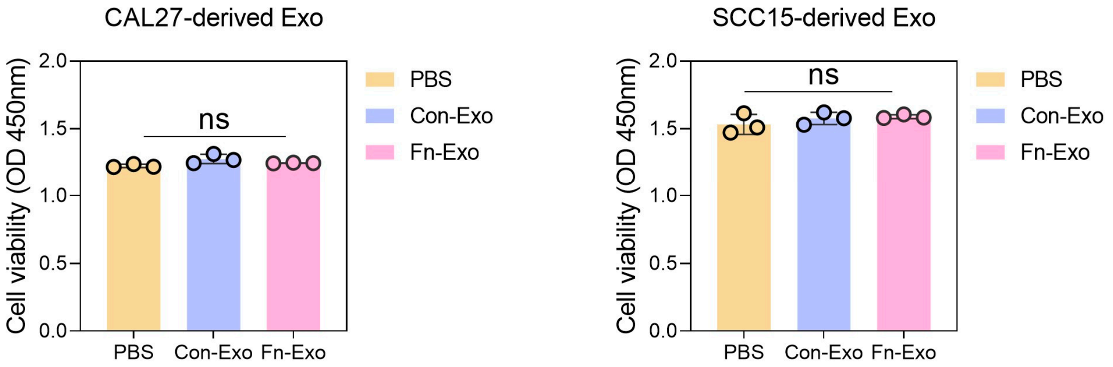

Figure S7. LEC viability following treatment with PBS, Con-Exo, or Fn-Exo, as assessed by CCK-8 assay. Data are presented as mean ± SD from three independent biological replicates. Statistical significance was determined by one-way ANOVA. ns, not significant.

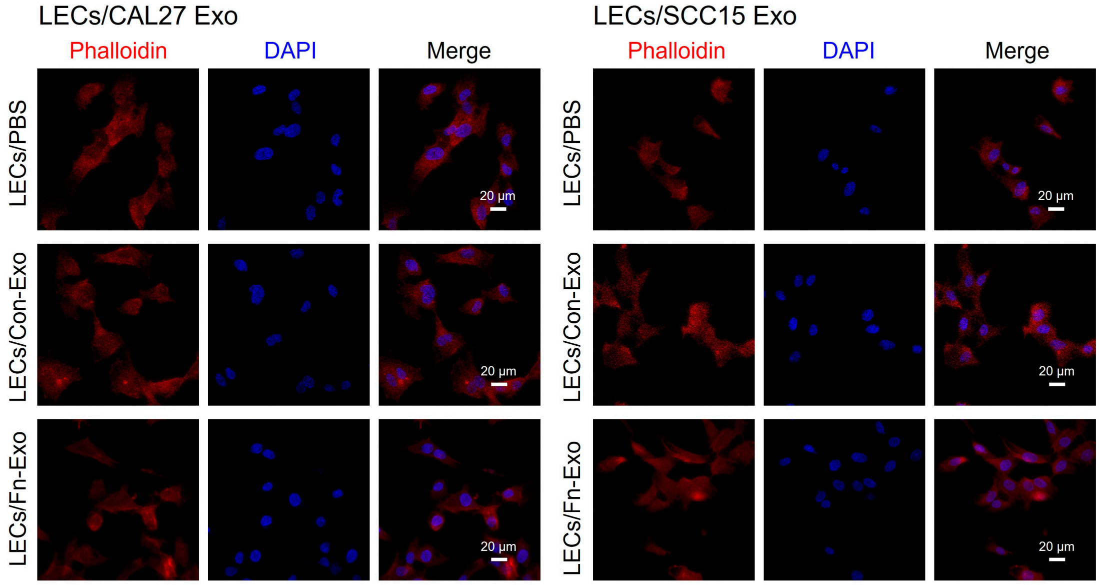

Figure S8. Morphological assessment of LECs following exosome treatment. Representative fluorescence images of LECs treated with PBS, Con-Exo, or Fn-Exo derived from CAL27 or SCC15 cells. F-actin was stained with phalloidin (red), and nuclei were counterstained with DAPI (blue). Scale bar, 20 μm.

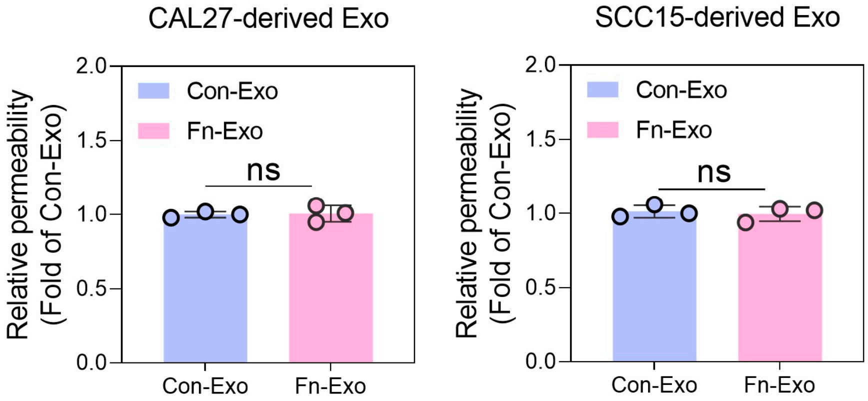

Figure S9. Endothelial permeability following exosome treatment. Relative permeability of LEC monolayers treated with CAL27- or SCC15-derived Con-Exo and Fn-Exo was assessed by FITC-dextran flux assay. Statistical significance was assessed by two-tailed unpaired Student's t-test. Data are shown as mean ± SD (n = 3). ns, not significant.

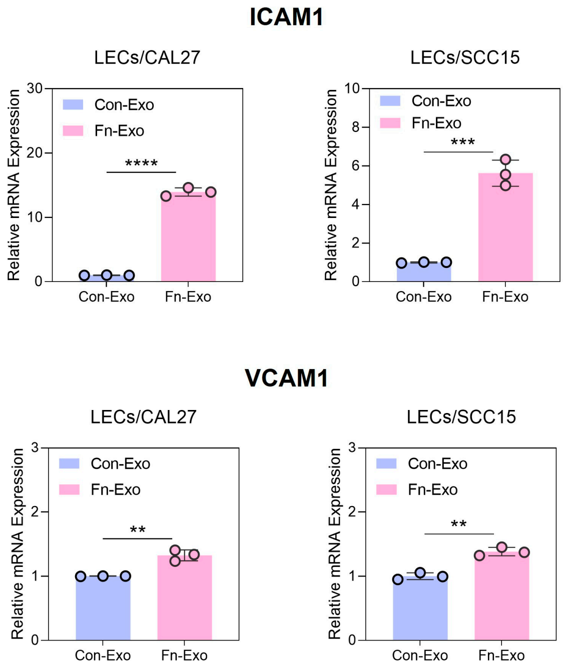

Figure S10. Expression of ICAM1 and VCAM1 in LECs following treatment with OSCC-derived exosomes. Relative mRNA expression of ICAM1 and VCAM1 in LECs treated with Con-Exo or Fn-Exo derived from CAL27 or SCC15 cells. Data are shown as mean ± SD (n = 3). Statistical significance was assessed by two-tailed unpaired Student's t-test. $^{**}P < 0.01$, $^{***}P < 0.001$, $^{**}P < 0.0001$.

Assessment of LEC viability, morphology, and permeability

LEC viability was assessed using a Cell Counting Kit-8 (Beyotime, China) according to the manufacturer's instructions. For morphological assessment, LECs were fixed with 4% paraformaldehyde for 10 min, permeabilized with 0.1% Triton X-100 for 10 min, and stained with red fluorescent phalloidin (YEASEN, China) diluted 1:200 for 1 h at room temperature in the dark. Nuclei were counterstained with DAPI (Solarbio, China), and fluorescence images were acquired using a fluorescence microscope. Endothelial permeability was assessed using a Transwell-based FITC-dextran flux assay. LECs were seeded onto 0.4 μm pore-size polycarbonate Transwell inserts (Corning, Corning, NY, USA) and cultured to form confluent monolayers. After the indicated exosome treatments, FITC-dextran (10 kDa; Beyotime, China; ST2935) was added to the upper chamber. After incubation for 60 min, fluorescence of the tracer that passed into the lower chamber was measured at an excitation wavelength of 494 nm and an emission wavelength of 521 nm.

Table S1 Primer sequences used for RT-qPCR.

**Gene**	**5′–3′ forward**	**5′–3′ reverse**
GAPDH	5’-GTGAAGGTCGGAGTCAACGG-3’	5’-GCAACAATATCCACTTTACCAGAGT-3’
TLR2	5’-ACCGTTTCCATGGCCTGTG-3’	5’-GATGTTCCTGCTGGGAGCTTTC-3’
RELB	5’-GCCCGTCTATGACAAGAAATCCA-3’	5’-CCTGCTGAACACCACTGATATG-3’
IL6	5’-CCAGAGCTGTGCAGATGAGTA-3’	5’-GTCCTGCAGCCACTGGTTC-3’
PTGS2	5’-CCAGCACTTCACGCATCAGTT-3’	5’-TGTCTAGCCAGAGTTTCACCGTAA-3’
TNFAIP3	5’-AAATCCCCGTCCAAGGCTG-3’	5’-CTGCTCGCTGTTTTCCTGC-3’
ICAM1	5’-TGTATGAACTGAGCAATGTGCAAGA-3’	5’-CACCTGGCAGCGTAGGGTAA-3’
VCAM1	5’-CGAAAGGCCCAGTTGAAGGA-3’	5’-GCACGAGAAGCTCAGGAGAAA-3’
